# A Systematic Review and Meta-Analysis of RCTs Assessing Efficacy of Lifestyle Interventions on Glycemic Control in South Asian Adults with Type 2 Diabetes

**DOI:** 10.3390/medsci14010048

**Published:** 2026-01-17

**Authors:** Ishtiaq Ahmad, Hira Taimur, Gowtham Venu Poduri, Allah Nawaz, Yoshihisa Shiriyama, Sameera Shabbir, Md. Shafiur Rahman, Aida Uzakova, Hafiz Sultan Ahmad, Miyoko Okamoto, Motoyuki Yuasa

**Affiliations:** 1 Department of Global Health Research, Graduate School of Medicine, Juntendo University, Tokyo 113-8421, Japan; 2 Faculty of Medicine, International Higher School of Medicine, Bishkek 720054, Kyrgyzstan; 3 Joslin Diabetes Centre, Harvard Medical School, Boston, MA 02215, USA; 4 School of Health Innovation, Kanagawa University of Human Services, Yokosuka 238-8522, Japan; 5 Faculty of Science and Technology, University of Central Punjab, Lahore 54000, Pakistan

**Keywords:** HbA1c, FBG, T2DM, lifestyle interventions, South Asia, meta-analysis

## Abstract

Background/Objective: The rising prevalence of Type 2 Diabetes Mellitus (T2DM), coupled with sedentary behavior and an increase in obesity rates in South Asian countries, calls for effective management strategies. We aimed to assess the efficacy of lifestyle interventions on glycemic control among adults with T2DM in South Asian countries. Methods: A systematic review and meta-analysis of randomized controlled trials (RCTs) were conducted to assess the effectiveness of lifestyle interventions on glycemic control in adults diagnosed with T2DM in South Asia. We conducted a comprehensive search in CINAHL, Embase, PubMed, Cochrane Library, Web of Science (WoS), and Scopus to identify related studies published from 2000 to 13 June 2024. We assessed the risk of bias using the ROB 2.0 tool and calculated the pooled mean differences in HbA1c and FBG levels under a random-effects model. We conducted subgroup and leave-one-out sensitivity analyses to assess and explore sources of heterogeneity. PROSPERO Registration: CRD42024552286. Results: We included 16 RCTs with a total of 1499 participants. Lifestyle interventions reduced HbA1c levels by 0.86% (95% CI: −1.30 to −0.42, *p* < 0.01) and FBG levels by 22.49 mg/dL (95% CI: −32.88 to −12.10, *p* < 0.01). We observed substantial heterogeneity (I^2^ = 98% for HbA1c and I^2^ = 87% for FBG). Subgroup analyses indicated larger HbA1c reductions in long-term (−1.44%) than short-term trials (−0.62%), and greater FBG decreases in long-term (−23.7 mg/dL) versus short-term studies (−22.5 mg/dL). Physical activity interventions had the largest improvements (HbA1c −0.99%; FBG −26.1 mg/dL), followed by dietary (HbA1c −0.59%; FBG −15.8 mg/dL) and combined programs (HbA1c −0.55%). Participants aged >50 years achieved greater glycemic improvements (HbA1c −0.92%; FBG −24.0 mg/dL) compared to younger adults (HbA1c −0.60%; FBG −21.3 mg/dL). Despite high heterogeneity, sensitivity analyses confirmed the robustness of the overall findings. Conclusions: Lifestyle modifications yielded a clinically significant reduction in HbA1c and FBG in adults with T2DM in South Asia. Although heterogeneity of the included studies was substantial, the direction of the effects was uniformly consistent across subgroups. To further validate these findings and assess their long-term effects, large-scale and standardized RCTs conducted for longer durations are necessary.

## 1. Introduction

Globally, Type 2 Diabetes Mellitus (T2DM) continues to rank among the most prevalent noncommunicable diseases (NCDs), affecting people across all age groups and genders [1]. According to the International Diabetes Federation Diabetes Atlas (11th Edition, 2025), T2DM and its complications contributed to an estimated 3.4 million deaths worldwide in 2024. indicating its substantial and growing contribution to global mortality [2]. In the South Asian populations, this burden is even greater due to genetic susceptibility and rapidly evolving lifestyle patterns [3]. Lifestyle changes play an important role in both the management and prevention of T2DM. Evidence shows that lifestyle interventions can bring down the T2DM incidence by 58% in high-risk groups of the population [4] and are effective across diverse racial and ethnic groups [5]. Key components like exercise and physical activity have been shown to play a vital role in improving glycemic control by lowering Hemoglobin A1C (HbA1c) and fasting blood glucose (FBG), among other health metrics [6].

The prevalence of T2DM in South Asia is mainly influenced by urbanization and a shift to a sedentary lifestyle, as well as a rapid dietary transition [7]. This shift includes a reduced consumption of coarse cereals, vegetables, and fruits, along with an increased carbohydrate intake [8,9]. The combination of low fiber, high glycemic index, and saturated fat in the diet contributes to poor glycemic control [10]. Personalized dietary interventions demonstrated significant improvements in HbA1c and FBG levels, highlighting the significance of individualized dietary management and nutrition education [11].

In spite of such established benefits, lifestyle changes are not consistently part of diabetes management in South Asia [12]. Because of this, many patients do not meet recommended treatment targets for HbA1c, blood pressure, or lipids [13]. The majority of studies on lifestyle interventions have focused on South Asians who are living abroad. However, diabetes management is shaped by more than genetic predisposition; it is also influenced by country-specific socio-economic, nutritional, and cultural factors [14], which makes it challenging to apply existing research findings. Although lifestyle modifications and dietary changes have shown effective results on glycemic control, the specific impact of these interventions in South Asian populations, where the burden of T2DM is particularly high, has not been fully established. Given the unique challenges in this region, more research is needed to determine the effectiveness of these interventions in South Asia. Therefore, we aimed to systematically review, assess, and consolidate evidence on lifestyle modifications, physical activity, dietary changes, or a combination of both to improve glycemic control among South Asian adults with T2DM.

## 2. Materials and Methods

We conducted a systematic review and meta-analysis of RCTs that quantified the impact of lifestyle modification interventions on glycemic control among patients with T2DM in South Asia. This review was registered in the International Prospective Register of Systematic Reviews (PROSPERO 2024 CRD42024552286) and conducted in accordance with the Preferred Reporting Items for Systematic Reviews and Meta-analyses guidelines [15,16].

### 2.1. Search Strategy

We conducted a comprehensive database search to identify relevant studies published between 1 January 2000 and 13 June 2024 across CINAHL, Embase, Cochrane Library, PubMed, Web of Science (WoS), and Scopus. We used the following keywords: (Exercise OR Diet OR Lifestyle OR “Lifestyle Interventions”) and (Sri Lanka, India, Pakistan, Bhutan, Nepal, Bangladesh, Maldives, South Asia) and Type 2 Diabetes. Appropriate Mesh terms and subject headings were used in search queries. We applied filters to refine the search, focusing on study design, randomized controlled trials (RCTs), English language, and free full-text access. In addition, references and citations of all eligible studies were searched. A detailed search strategy for each database can be found in [Supplementary Tables S1–S6].

### 2.2. Study Selection and Data Extraction

Records from all the databases were combined, and duplicate titles were removed using EndNote 21 [17]. Two reviewers (IA and GP) independently screened titles and abstracts, and subsequently retrieved full texts to assess the eligibility for inclusion. We resolved the disagreements about eligibility through discussion, and consensus was reached in all cases. Two reviewers independently extracted the following information from the finally selected papers: author, study type, study location, length of the study, and characteristics of the participants (sample size, age, and gender) in the intervention group and control group. The type of intervention, post-intervention mean values, and standard deviations were also extracted.

### 2.3. Eligibility Criteria

Studies were included if (a) it was conducted among adults aged 18 years and older in South Asia who were clinically diagnosed with Type 2 Diabetes Mellitus; (b) the study design was RCTs with a comparison group (e.g., standard care, no intervention); (c) lifestyle interventions that consist of structured and planned programs involving dietary modification, physical activity, or a combination of both; and (d) the outcome was HbA1c levels or fasting blood glucose (FBG) levels. However, studies were excluded if they involved participants under 18 years of age, individuals without a T2DM diagnosis, or pregnant or lactating women. In addition, we excluded the studies if they were not written in English, conducted on non-human populations, or published as conference abstracts, review articles, opinion pieces, or letters to the editor.

### 2.4. Outcomes

The outcome of interest was the change in glycemic control, as measured by HbA1c (%) (Glycated Hemoglobin) or fasting blood glucose (FBG) (mg/dL).

### 2.5. Risk of Bias

One author (I.A) assessed the risk of bias, and a second author (G.V.*p*) reviewed it using the RoB 2.0 tool by Cochrane [18]. This assessment considered aspects such as bias arising from the randomization process, deviations from intended interventions, missing outcome data, measurement of the outcome, and selection of the reported result

Each domain was rated as low risk of bias, high risk of bias, and some concerns. As specified in the Cochrane Handbook for Systematic Reviews to assess the risk of bias [19], if we determine that any of the domains has a high risk of bias in a trial, then the overall risk of bias is considered high. Any disagreements were resolved by consensus.

### 2.6. Effect Sizes

The effect size of interest was the mean difference (MD). We extracted post-intervention data on HbA1c and/or FBG. We extracted the mean and standard deviation (SD) for the HbA1c or FBG values in both the intervention and control groups. The ESs were estimated using the MD of post-intervention HbA1c/FBG values in both intervention and control groups.

### 2.7. Data Analysis

A random-effects meta-analysis was conducted to estimate the pooled mean difference for two clinical outcomes—HbA1c and FBG. We assessed the level of heterogeneity (I2 statistic) between studies. We also conducted subgroup analyses based on duration of intervention, participants’ age, and type of lifestyle modification: dietary, physical activity, and combined (both physical activity and dietary). We performed a leave-one-out sensitivity analysis to explore the influence of each included study on the pooled ES. We assessed the publication bias by visually examining the asymmetry in funnel graphs, Egger’s test, and Begg’s test, and we addressed the bias using the Trim and Fill method. Statistical analyses were performed in R Version 4.4.1 [20] using the “metafor” [21] and “meta” [22] packages

## 3. Results

**Figure 1:**
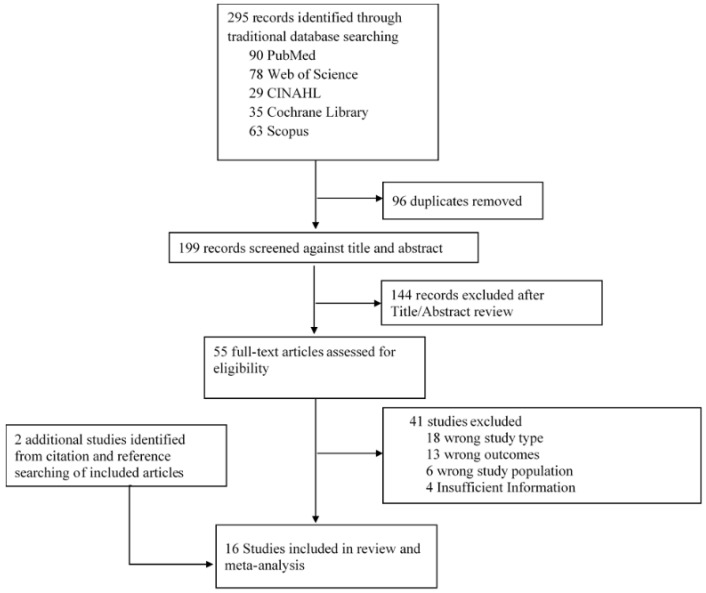
PRISMA flow diagram.

Our initial search identified 295 unique studies that are potentially eligible for inclusion in the meta-analysis. After reviewing the titles and abstracts, we excluded several. We assessed 55 full-text articles, and 16 Studies met the inclusion criteria for our review and meta-analysis. Additionally, we searched for references and citations from eligible articles and included any further studies that met the inclusion criteria. A PRISMA flow diagram summarizing this process is presented in [Figure 1].

All interventions in the included studies were integrated into the context of usual care. The type of lifestyle intervention differed between studies; 10 studies [23,24,25,26,27,28,29,30,31,32] focused solely on physical activity, 4 studies [33,34,35,36] implemented dietary changes exclusively, and 2 studies [37,38] incorporated both physical activity and dietary components. The interventions differed significantly in duration, intensity, delivery method, and focus. On average, the interventions lasted about 5 months, with durations ranging from 2 to 12 months [30,35]. A total of eleven studies [23,24,25,27,28,29,31,32,33,37,38] employed short-term interventions (less than six months), while five studies [26,30,34,35,36] employed longer-term interventions (six months or more). Participant age varied across studies; eleven studies [23,24,28,29,30,31,32,33,34,35,37] predominantly involved older adults (average age 50+), while five studies [25,26,27,36,38] included participants with an average age below 50 years.

Table 1 summarizes the key characteristics of the clinical trials included in this review. Across the included studies, sample sizes ranged from 18 to 117 participants. The studies were conducted in South Asian countries and varied in design, encompassing RCTs, prospective cohort studies, and case–control designs. The duration of follow-up ranged from 2 to 12 months, allowing for comprehensive assessment of both short- and long-term outcomes.

**Figure 2:**
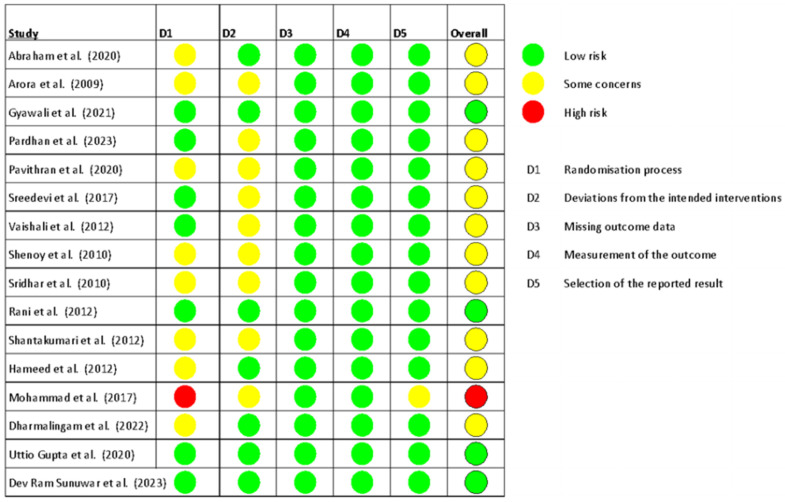
Risk of bias in all trials included in the review [23,24,25,26,27,28,29,30,31,32,33,34,35,36,37,38].

**Figure 3:**
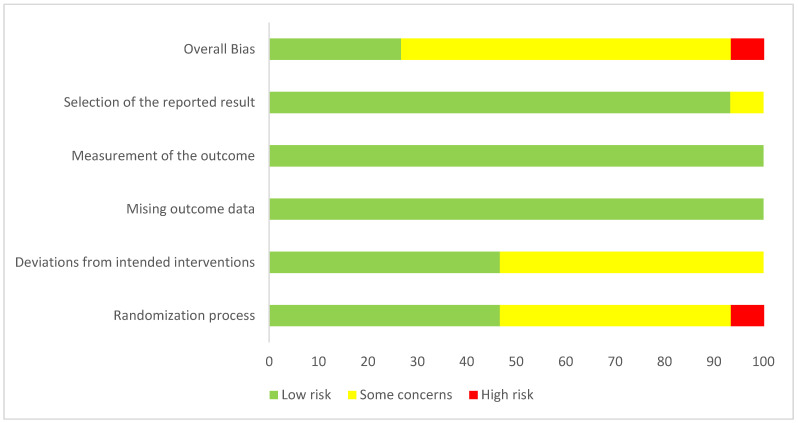
Overall risk of bias summary of RCTs included in the meta-analyses.

In terms of risk of bias, most RCTs were rated as having some concerns (*n* = 11, 69%), followed by low risk (*n* = 4, 25%), and high risk (*n* = 1, 6%). The risk of bias for all included trials is presented in [Figure 2], and the percentage of trials categorized as high risk, low risk, or with some concerns is detailed in [Figure 3].

**Figure 4:**
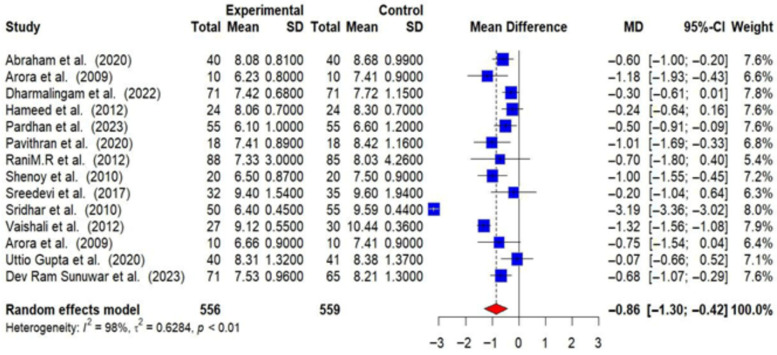
Meta-analysis of the post-intervention mean difference in HbA1c between the intervention and control groups [23,24,26,28,29,30,31,32,33,34,36,37,38]. Each blue square represents an individual study result, with larger squares indicating higher weight. The horizontal lines show the 95% confidence intervals. The red diamond represents the overall pooled effect from the random-effects model. The solid vertical line indicates no effect, and the dotted line shows the overall pooled estimate.

A total of 13 RCTs provided post-intervention data on HbA1c. The post-intervention pooled MD in HbA1c levels between intervention and control groups was −0.86% (95% CI: −1.30 to −0.42). This reduction is statistically significant (*p* < 0.01), indicating that lifestyle modifications effectively lower HbA1c levels among T2DM patients. However, substantial heterogeneity was observed among the studies (I^2^ = 98%), suggesting that the effectiveness of the interventions varied across different studies [Figure 4].

**Figure 5:**
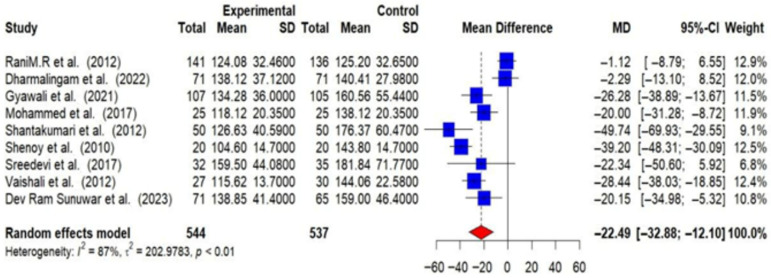
Meta-analysis of the post-intervention mean difference in FBG levels between intervention and control groups [25,26,27,28,29,31,33,35,36].

Out of 16 RCTs, 9 provided post-intervention data on FBG among T2DM patients. Using a random-effects meta-analysis, the post-intervention pooled MD in FBG levels between intervention and control groups was found to be −22.49 mg/dL (95% CI: −32.88 to −12.10), indicating that lifestyle modifications effectively lower FBG levels (*p* < 0.01). A substantial level of heterogeneity was observed among the studies (I^2^ = 87%), suggesting that the effectiveness of the interventions varied across different studies [Figure 5].

**Figure 6:**
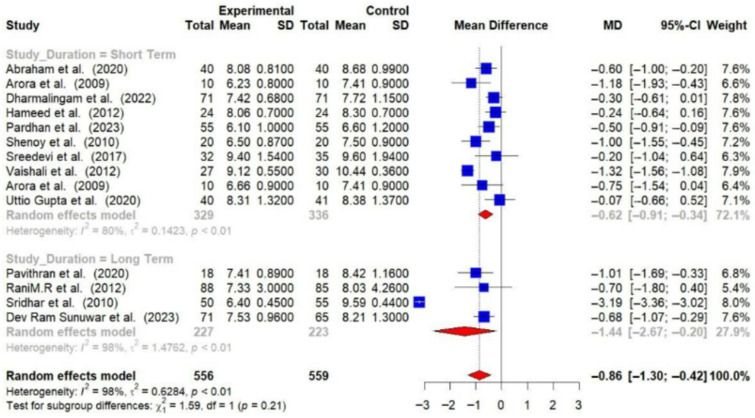
Meta-analysis of the post-intervention mean difference in HbA1c levels between intervention and control groups by the duration of study [23,24,26,28,29,30,31,32,33,34,36,37,38]. The grey color was automatically suggested by RStudio (2025.07.2+1299 “Ocean Storm”) program to differentiate the sub-group names from the author names. The color was not manually selected.

**Figure 7:**
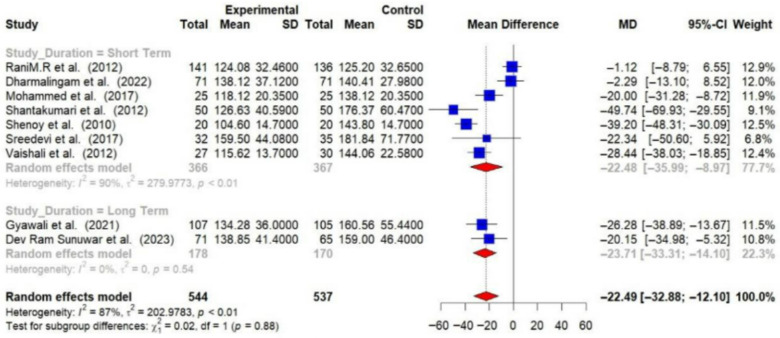
Meta-analysis of the post-intervention mean difference in FBG levels between intervention and control groups by the study duration [25,26,27,28,29,31,33,35,36]. The grey color was automatically suggested by RStudio program to differentiate the sub-group names from the author names. The color was not manually selected.

Subgroup analyses were conducted, and detailed forest plots are provided in the Supplementary Materials. Long-term trials (≥6 months) demonstrated a greater reduction in HbA1c (−1.44% [95% CI: −2.67 to −0.20]) and FBG (−23.71 mg/dL [95% CI: −33.31 to −14.10]) compared to short-term trials (<6 months) (HbA1c: −0.62% [95% CI: −0.91 to −0.34]; FBG: −22.48 mg/dL [95% CI: −35.99 to −8.97]) [Figure 6 and Figure 7].

**Figure 8:**
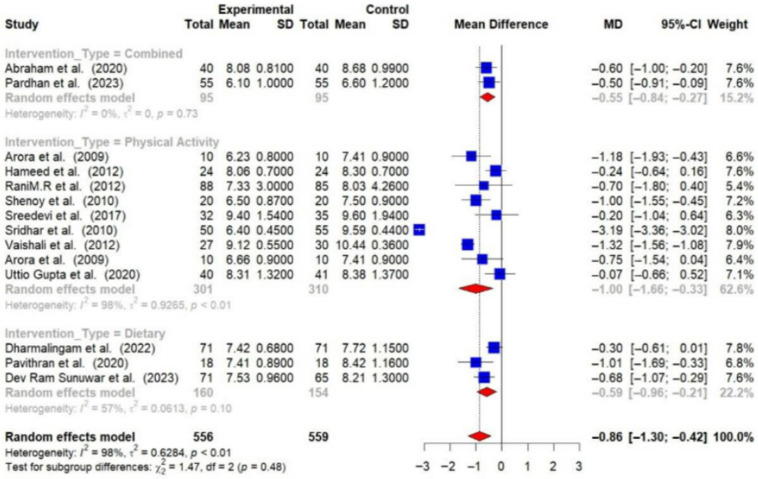
Meta-analysis of the post-intervention mean difference in HbA1c levels between intervention and control groups by the type of intervention [23,24,26,28,29,30,31,32,33,34,36,37,38]. The grey color was automatically suggested by RStudio program to differentiate the sub-group names from the author names. The color was not manually selected.

**Figure 9:**
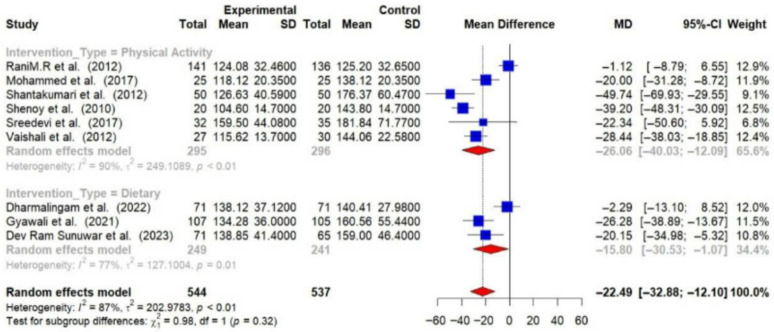
Meta-analysis of the post-intervention mean difference in FBG levels between intervention and control groups by the type of intervention [25,26,27,28,29,31,33,35,36]. The grey color was automatically suggested by RStudio program to differentiate the sub-group names from the author names. The color was not manually selected.

Physical activity interventions led to more substantial reductions in HbA1c (−0.99% [95% CI: −1.66 to −0.33]) and FBG (−26.06 mg/dL [95% CI: −40.03 to −12.09]) compared to dietary interventions (HbA1c: −0.59% [95% CI: −0.96 to −0.21]; FBG: −15.80 mg/dL [95% CI: −30.53 to −1.07]). Combined interventions also showed a decrease in HbA1c (−0.55 [95% CI: −0.84 to −0.27]) [Figure 8 and Figure 9].

**Figure 10:**
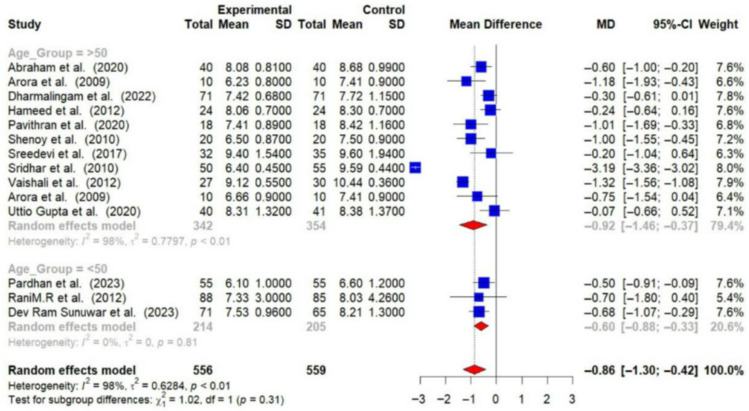
Meta-analysis of the post-intervention mean difference in HbA1c levels between the intervention and control groups by age group [23,24,26,28,29,30,31,32,33,34,36,37,38]. The grey color was automatically suggested by RStudio program to differentiate the sub-group names from the author names. The color was not manually selected.

**Figure 11:**
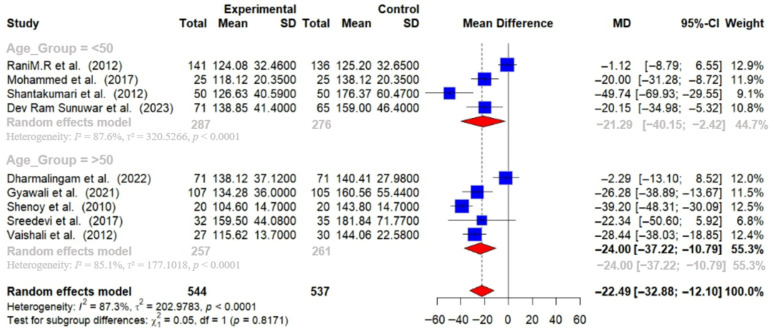
Meta-analysis of the post-intervention mean difference in FBG levels between intervention and control groups by age group [25,26,27,28,29,31,33,35,36]. The grey color was automatically suggested by RStudio program to differentiate the sub-group names from the author names. The color was not manually selected.

Participants over 50 years of age experienced greater reductions in HbA1c (−0.92% [95% CI: −1.46 to −0.37]) and FBG (−24.00 mg/dL [95% CI: −37.22 to −10.79]) compared to those under 50 years (HbA1c: −0.60% [95% CI: −0.88 to −0.33]; FBG: −21.29 mg/dL [95% CI: −40.15 to −2.42]) [Figure 10 and Figure 11].

Overall, these studies demonstrate that lifestyle interventions are more effective than dietary interventions in lowering HbA1c, with patients over 50 years showing greater improvements in both HbA1c and fasting blood glucose levels. Despite these observed differences in effect sizes across subgroups, none of the tests for subgroup differences were statistically significant for either HbA1c or FBG, and marked heterogeneity was present.

We found considerable heterogeneity in the pooled analyses (HbA1c: I^2^ = 98%; FBG: I^2^ = 87%). However, subgroup analyses revealed that heterogeneity was markedly reduced in certain categories. For example, combined lifestyle interventions showed perfect consistency (I^2^ = 0%) for HbA1c, while dietary interventions had moderate heterogeneity (I^2^ = 57%). Similarly, heterogeneity for FBG was not present in long-term studies (I^2^ = 0%) and reduced in dietary interventions (I^2^ = 77%).

We conducted a leave-one-out sensitivity analysis for both HbA1c and FBG to assess the robustness of the results. For HbA1c, the pooled MD ranged from −0.66 to −0.92, and heterogeneity remained high (I^2^ > 97%), an indication of the consistency in findings across the different studies. For FBG, the pooled MD ranged from −19.73 to −25.55, with heterogeneity also remaining high (I^2^>79%). These results imply that no single study had a disproportionate impact on the overall effect, confirming the stability of the findings [Supplementary Figures S1 and S2].

We assessed publication bias using Egger’s test, Begg’s test, and the trim-and-fill method. Egger’s test showed that there was significant funnel plot asymmetry for HbA1c (t = 3.02, *p* = 0.0106), indicating potential publication bias, though there was no significant asymmetry in FBG (t = −0.95, *p* = 0.37). Begg’s test did not show any publication bias for either HbA1c (Kendall’s tau = −0.25, *p* = 0.23) or FBG (Kendall’s tau = −0.56, *p* = 0.06). We used the trim-and-fill method to adjust for potential bias, which imputed additional studies. For HbA1c, this adjustment suggested that the intervention effect might be underestimated (MD = −2.09, 95% CI [−2.84; −1.34] vs. MD = −0.86, 95% CI [−1.30; −0.42] without adjustment). For FBG, the imputed studies resulted in a less negative mean difference (MD = −8.51, 95% CI [−21.34; 4.32] vs. MD = −22.49, 95% CI [−32.88; −12.09]), indicating potential overestimation of the intervention effect. Overall, our analysis suggests that while there is some evidence of publication bias in the HbA1c findings, it may not be substantial for FBG [Supplementary Figures S3–S6]. Egger’s and Begg’s tests suggested potential publication bias in studies assessing HbA1c; it may not be substantial for FBG, and the trim-and-fill method identified additional imputed studies, indicating that the overall effect estimates may be slightly overestimated.

## 4. Discussion

### 4.1. Glycemic Outcomes of Lifestyle Interventions

This systematic review and meta-analysis assessed the impact of lifestyle interventions on glycemic control in adults with T2DM across South Asian countries, focusing exclusively on RCTs. Our study offered a critical, region-specific evaluation of these interventions in the South Asian population, demonstrating a statistically and clinically significant reduction in both HbA1c and FBG levels.

The reduction of 0.5% of HbA1c is considered clinically significant [39]. Notably, we found a mean reduction of 0.86% in HbA1c and 22.49 mg/dL in FBG, which are larger than those reported in the meta-analyses by Jansson AK et al. [40], and Yuwen Wan et al. [41], This discrepancy in effect size may be attributed to the exclusive focus of our review on the South Asian population, which may have distinct genetic predispositions and responses to lifestyle interventions compared to the populations included in other reviews.

These findings are also comparable to the results of the Look AHEAD trial [42], which reported a 0.64% reduction in HbA1c after one year of intensive lifestyle modification among individuals with T2DM. Although the landmark diabetes prevention trials Finnish Diabetes Prevention Study [4] and USA Diabetes Prevention Program Research Group [5] did not report direct reductions in HbA1c or FBG, both demonstrated that structured lifestyle changes substantially lowered the incidence of T2DM.

### 4.2. Physical Activity and Glycemic Control

Exercise is an extremely important part of lowering blood sugar levels, achieved through multiple mechanisms. Both strength training and aerobic exercises promote the activity of the GLUT4 transporter protein in skeletal muscles, thus increasing the amount of glucose absorbed from the blood, thereby lowering blood sugar levels [43]. Physical activity also increases the sensitivity of skeletal muscle cells to insulin by increasing their oxidative capacity, the amount of mitochondria, and their reduced ability to accumulate excess lipid molecules [44]. Exercise also allows the entry of glucose into the cells despite low levels of insulin by stimulating the AMPK pathway [45].

### 4.3. Dietary Interventions and Glycemic Control

Similarly, a low-glycemic index and high fiber diet prevents a sudden rise in glucose levels [46]. Restriction of calorie intake and a balanced nutrient diet improve insulin sensitivity, thereby reducing hepatic glucose production, thus lowering the levels of fasting glucose and Hemoglobin A1C [47]. In addition, diets such as Mediterranean or low-carbohydrate diets improve lipid and inflammation status, therefore offering a positive impact on metabolism among diabetic patients [48].

### 4.4. Comparison of Physical Activity and Dietary Interventions

The majority of the included studies emphasized physical activity as the primary intervention, encompassing various forms such as yoga [25,26,27,29,31,32], resistance training [23,24], aerobic exercises, and daily exercise routines [28,30,38]. Physical activity interventions led to larger reductions in HbA1c (−0.99%) and FBG (−26.06 mg/dL) compared to dietary interventions (HbA1c: −0.59% and FBG: −15.80 mg/dL), indicating the vital role of physical activity in the management of T2DM patients. These results are consistent with the existing literature. For example, Jansson AK et al. [40] reported a significant reduction in HbA1c with resistance training (weighted MD = −0.39%), while Yuwen Wan et al. [41] observed reductions in HbA1c (−0.50%) and FBG (−12.03 mg/dL). However, these reviews have included studies of different populations, potentially explaining the relatively smaller effect size as compared to our findings in South Asian adults. In contrast, Jun Jia et al. [49] found that resistance training interventions did not significantly change HbA1c levels compared to controls (−0.22%, 95%CI: −0.98 to 0.54). They suggested that their focus was on quality of life rather than glycemic control, which may have influenced these findings, emphasizing the need for targeted interventions when assessing HbA1c outcomes.

Dietary interventions, while fewer in number, also played a role in improving glycemic control. Consistent with Parisa Ghasemi et al. [50] which demonstrated reductions in FBG (−11.68 mg/dL) and HbA1c (−0.29%) with a very low-carbohydrate ketogenic diet, our results (HbA1c: −0.59% [95% CI: −0.96 to −0.21]; FBG: −15.80 mg/dL [95% CI: −30.53 to −1.07]) support the role of dietary modifications in improving glycemic control through improved nutritional literacy. Encouraging dietary changes at both clinical and community levels can improve awareness and adherence to diabetes-specific nutritional guidelines, which in turn leads to better patient outcomes.

### 4.5. Heterogeneity and Subgroup Analyses

We observed high heterogeneity in our pooled results, which might have resulted from differences in lifestyle interventions, duration of the studies, and participant characteristics within the South Asian studies. When we performed a subgroup analysis, we found that some of this variability could be explained by these factors. For example, studies that used combined lifestyle interventions and those with longer follow-up periods showed more consistency in the results. Overall, even though the heterogeneity across studies was high, the direction of the effect consistently favored lifestyle interventions for improving glycemic control.

### 4.6. Strengths and Limitations

The strengths of our study are that we only included RCTs, which have long been recognized as the gold standard for evidence [51], and the comprehensive nature of our analysis, which included random-effects meta-analysis, subgroup analysis, sensitivity analysis, and assessment of publication bias. To the best of our knowledge, our systematic review and meta-analysis are the first to specifically investigate the impact of lifestyle interventions on glycemic control in South Asian adults with T2DM.

However, some limitations must be acknowledged. Most studies were carried out in India, with only a limited number of studies from other South Asian countries, limiting the generalizability of our findings to the entire South Asian population. The short-term nature of follow-up data restricts our ability to determine the sustained impact of lifestyle interventions. Another limitation was that we were unable to directly apply Egger’s test for FBG due to low sample size (*n* = 9), which did not meet the minimum threshold of 10 required for reliable results. Because of this, we had to modify the standard procedure. Therefore, we conducted the Begg’s test and used the trim-and-fill method to assess potential bias.

### 4.7. Conclusion and Implications

The results showed clinically significant evidence that lifestyle interventions can improve glycemic control in adults with T2DM in South Asia. Despite the high heterogeneity, there has been a consistent reduction in HbA1c and FBG throughout the subgroups. These findings align with existing international guidelines from the IDF, American Diabetes Association (ADA) [2,52], and the World Health Organization (WHO) [53], all of which recommend lifestyle modifications like regular physical activity and dietary changes as crucial components of diabetes management and prevention. Future research should prioritize multi-center, well-supervised RCTs with longer follow-up periods in diverse South Asian populations. This is because participants in well-supervised studies are more likely to experience greater positive outcomes due to the structured guidance and consistent support provided throughout all sessions of the study [54]. Along with this, national guidelines should integrate dietary interventions and moderate physical activity into standard diabetic care at the health center level, along with initiatives to raise awareness and improve nutritional literacy among South Asian T2DM patients.

## Figures and Tables

**Table 1:** Characteristics of included trials.

S. No	Author (Year)	Study Location	Length of the Study	Sample size of the Participants in the Intervention Group	Mean Age of Intervention Group	Sample Size of the Participants in the Control Group	Mean Age of the Control Group	Intervention Type	Control Group	Outcome Measures	Overall Risk of Bias
1	Abraham et al. (2020) [37]	Bangalore, India	3 months	*n* = 40	50.4	*n* = 40	50.6	Combined: Sessions on Diet, Exercise, Diabetes Education	Usual Care	HbA1c	Some Concerns
2	Arora et al. (2009) [23]	Amritsar, India	8 Weeks	*n* = 20	50.9	*n* = 10	58.4	Physical Activity: Progressive Resistance Training and Aerobic Exercise	Usual Care	HbA1c	Some Concerns
3	Dharmalingam et al. (2022) [33]	Across five centers in India: Bangalore, Guwahati, Jaipur, Ahmedabad, and Kolkata	12 weeks	*n* = 85	50.44	*n* = 86	49.92	Dietary: Partial Meal Replacement, Dietary Counseling, Diet Charts	Usual Care	HbA1c, FBG	Some Concerns
4	Gyawali et al. (2021) [35]	Semiurban areas in Western Nepal	12 months	*n* = 127	51.71	*n* = 117	52.29	Dietary: FCHV-led lifestyle intervention (home visits, counseling about dietary habits and nutrition, monitoring)	Usual Care	FBG	Low
5	Hameed et al. (2012) [24]	New Delhi, India	8 weeks	*n* = 24	45.0	*n* = 24	44.3	Physical Activity: Progressive Resistance training	Usual care	HbA1c	Some Concerns
6	Mohammad et al. (2017) [25]	Mysuru, India	4 months	*n* = 25	41.27	*n* = 25	40.28	Physical Activity Yoga (1 h/day)	Usual Care	FBG	High
7	Pardhan et al. (2023) [38]	Pokhara, Nepal	3 months	*n* = 55	45	*n* = 55	47	Combined: Culturally appropriate video education, customized diet, and exercise plan	Usual Care	HbA1c	Some Concerns
8	Pavithran et al. (2020) [34]	Kerala, India	6 months	*n* = 18	52.77	*n* = 18	52.77	Dietary: Low GI Diet	Usual Care	HbA1c, FBG	Some Concerns
9	Rani et al. (2012) [26]	Bengaluru, India	9 months	*n* = 141	53.4	*n* = 136	51.3	Physical Activity Yoga-based lifestyle modification program (YLSP)	Usual Care	HbA1c FBG	Low
10	Shantakumari et al. (2012) [27]	Trivandrum, Kerala, India	3 months	*n* = 50	45.51	*n* = 50	44.46	Physical Activity: Yoga	Usual care	FBS,	Some Concerns
11	Shenoy et al. (2010) [28]	Amritsar, India	8 weeks	*n* = 20	53.15	*n* = 20	51	Physical Activity: Aerobic Walking using HRM and Pedometer	Usual Care	HbA1c, FBG	Some Concerns
12	Sreedevi et al. (2017) [29]	Kerala, India	3 months	*n* = 35	51.9	*n* = 38	51.9	Physical Activity Yoga	Usual Care	FBG, HbA1C	Some Concerns
13	Sridhar et al. (2010) [30]	Mangalore, India	12 months	*n* = 55	61	*n* = 50	59	Physical Activity: Regular exercise training	Usual Care	HbA1C	Some Concerns
14	Vaishali et al. (2012) [31]	Mangalore, India	3 months	*n* = 27	65.8	*n* = 30	64.4	Physical Activity: Yoga-based program	Usual Care	HbA1C, FBG	Some Concerns
15	Uttio Gupta et al. (2020) [32]	New Delhi, India	4 months	*n* = 40	51.1	*n* = 41	50.2	Physical Activity: Yoga-based exercise program	Usual Care	HbA1c	Low
16	Dev Ram Sunuwar et al. (2023) [36]	Nepal	6 months	*n* = 78	46.7	*n* = 78	50.3	Dietary: Customized Diet Plan	Usual Care	HbA1c, FBG	Low

Note: FCHV—female community health volunteers; GI—glucose index; YLSP—yoga-based lifestyle modification program; HRM—heart rate monitor; FBG—fasting blood glucose.

## Data Availability

No new data were created or analyzed in this study.

## Supplementary Materials

The following supporting information can be downloaded at: https://www.mdpi.com/article/10.3390/medsci14010048/s1, Table S1: PubMed Search Strategy, Table S2: Web Of Science Search Strategy, Table S3: Scopus search Strategy, Table S4: CINAHL search Strategy (In EBSCOHost), Table S5: Cochrane Library Search Strategy, Table S6: Summary Results of database search and full-text screening, Figure S1: Sensitivity Analyses for HbA1c by performing the meta-analysis by excluding one study at a time, Figure S2: Sensitivity Analyses for FBG by performing the meta-analysis by excluding one study at a time, Figure S3: Funnel plot for random effects meta-analysis of post intervention mean difference of HbA1C Levels, Figure S4: Funnel plot for random effects meta-analysis of post intervention mean difference of FBG Levels, Figure S5: Funnel and Forest plots Publication Bias Analysis using Trim and Fill method for mean difference of HbA1c levels, Figure S6: Funnel and Forest plots of Publication Bias Analysis using Trim and Fill method for mean difference of FBG levels, PRISMA 2020 Checklist
