# Supplementary material for: A Systematic Review and Meta-Analysis of RCTs Assessing Efficacy of Lifestyle Interventions on Glycemic Control in South Asian Adults with Type 2 Diabetes
Source: Med Sci (Basel). 2026 Jan 17;14(1):48. doi: 10.3390/medsci14010048 (PMC12821424; doi:10.3390/medsci14010048)
Supplement: Supplementary file 1 [file medsci-14-00048-s001.zip › medsci-4048334-supplementary.pdf]

Supplementary Appendix

A Systematic Review and Meta-Analysis of RCTs Assessing Efficacy of Lifestyle Interventions on Glycemic Control in South Asian Adults with Type 2 Diabetes

Ishtiaq Ahmad [1,*], Hira Taimur [1], Gowtham Venu Poduri [2], Allah Nawaz [3], Yoshihisa Shiriyama [1], Sameera Shabbir [3], Md. Shafiur Rahman [4], Aida Uzakova [2], Hafiz Sultan Ahmad [5], Miyoko Okamoto [1] and Motoyuki Yuasa [1]

[1] Department of Global Health Research, Graduate School of Medicine, Juntendo University, Tokyo 113-8421, Japan; h.taimur.yv@juntendo.ac.jp (H.T.); shirayam@juntendo.ac.jp (Y.S.); myokamo@juntendo.ac.jp (M.O.); moyuasa@juntendo.ac.jp (M.Y.)

[2] International Higher School of Medicine, Bishkek, 720054 Kyrgyzstan; gowtham.poduri@gmail.com (G.V.P.); aidauzakovaa@gmail.com (A.U.); drsameeraasad@gmail.com (S.S.)

[3] Joslin Diabetes Centre, Harvard Medical School, Boston, MA, USA; allah.nawaz@joslin.harvard.edu (A.N.);

[4] School of Health Innovation, Kanagawa University of Human Services, Yokosuka, Japan; rahman-2s5@kuhs.ac.jp

[5] Faculty of Science and Technology, University of Central Punjab, Lahore, Pakistan; sultanrajpoot65@gmail.com

* Correspondence: ahmad@juntendo.ac.jp

Index

Search strategy for the systematic literature search used on PubMed, Cochrane Library, Web of Science, Scopus, CINAHL 2

Supplementary Table S1: PubMed Search Strategy 2

Supplementary Table S2 : Web Of Science Search Strategy 4

Supplementary Table S3: Scopus search Strategy 4

Supplementary Table S4: CINAHL search Strategy (In EBSCOHost) 4

Supplementary Table S5: Cochrane Library Search Strategy 4

Supplementary Table S6: Summary Results of database search and full-text screening 5

Inclusion and exclusion criteria 6

Sub-group analyses for HbA1c 6

Sensitivity Analysis 6

Supplementary Figure S2: Sensitivity Analyses for FBG by performing the meta-analysis by excluding one study at a time 7

Supplementary Figure S3: Funnel plot for random effects meta-analysis of post intervention mean difference of HbA1C Levels 7

Supplementary Figure S4: Funnel plot for random effects meta-analysis of post intervention mean difference of FBG Levels 7

Supplementary Figure S5: Funnel and Forest plots Publication Bias Analysis using Trim and Fill method for mean difference of HbA1c levels 8

Supplementary Figure S6: Funnel and Forest plots of Publication Bias Analysis using Trim and Fill method for mean difference of FBG levels 9

Search strategy for the systematic literature search used on PubMed, Cochrane Library, Web of Science, Scopus, CINAHL

Supplementary Table S1: PubMed Search Strategy

Country	Query	Search Details	Results
Maldives	(Exercise OR Diet OR Lifestyle OR "Lifestyle Interventions") AND Maldives AND Diabetes Mellitus AND ((randomizedcontrolledtrial[Filter]) AND (2000:2024[pdat]))	(("exercise"[MeSH Terms] OR "exercise"[All Fields] OR "exercises"[All Fields] OR "exercise therapy"[MeSH Terms] OR ("exercise"[All Fields] AND "therapy"[All Fields]) OR "exercise therapy"[All Fields] OR "exercising"[All Fields] OR "exercise s"[All Fields] OR "exercised"[All Fields] OR "exerciser"[All Fields] OR "exercisers"[All Fields] OR ("diet"[MeSH Terms] OR "diet"[All Fields]) OR ("life style"[MeSH Terms] OR ("life"[All Fields] AND "style"[All Fields]) OR "life style"[All Fields] OR "lifestyle"[All Fields] OR "lifestyles"[All Fields]) OR "Lifestyle Interventions"[All Fields]) AND ("maldive"[All Fields] OR "maldives"[MeSH Terms] OR "maldives"[All Fields]) AND ("diabetes mellitus"[MeSH Terms] OR ("diabetes"[All Fields] AND "mellitus"[All Fields]) OR "diabetes mellitus"[All Fields]) AND ("randomized controlled trial"[Publication Type] AND 2000/01/01:2024/12/31[Date - Publication])) AND ((ffrft[Filter]) AND (randomizedcontrolledtrial[Filter]))	0
Bhutan	(Exercise OR Diet OR Lifestyle OR "Lifestyle Interventions") AND Bhutan AND Diabetes Mellitus AND ((randomizedcontrolledtrial[Filter]) AND (2000:2024[pdat]))	(("exercise"[MeSH Terms] OR "exercise"[All Fields] OR "exercises"[All Fields] OR "exercise therapy"[MeSH Terms] OR ("exercise"[All Fields] AND "therapy"[All Fields]) OR "exercise therapy"[All Fields] OR "exercising"[All Fields] OR "exercise s"[All Fields] OR "exercised"[All Fields] OR "exerciser"[All Fields] OR "exercisers"[All Fields] OR ("diet"[MeSH Terms] OR "diet"[All Fields]) OR ("life style"[MeSH Terms] OR ("life"[All Fields] AND "style"[All Fields]) OR "life style"[All Fields] OR "lifestyle"[All Fields] OR "lifestyles"[All Fields]) OR "Lifestyle Interventions"[All Fields]) AND ("bhutan"[MeSH Terms] OR "bhutan"[All Fields] OR "bhutan s"[All Fields]) AND ("diabetes mellitus"[MeSH Terms] OR ("diabetes"[All Fields] AND "mellitus"[All Fields]) OR "diabetes mellitus"[All Fields]) AND ("randomized controlled trial"[Publication Type] AND 2000/01/01:2024/12/31[Date - Publication])) AND ((ffrft[Filter]) AND (randomizedcontrolledtrial[Filter]))	0

Nepal	(Exercise OR Diet OR Lifestyle OR "Lifestyle Interventions") AND Nepal AND Diabetes Mellitus AND ((randomizedcontrolledtrial[Filter]) AND (2000:2024[pdat]))	(("exercise"[MeSH Terms] OR "exercise"[All Fields] OR "exercises"[All Fields] OR "exercise therapy"[MeSH Terms] OR ("exercise"[All Fields] AND "therapy"[All Fields]) OR "exercise therapy"[All Fields] OR "exercising"[All Fields] OR "exercise s"[All Fields] OR "exercised"[All Fields] OR "exerciser"[All Fields] OR "exercisers"[All Fields] OR ("diet"[MeSH Terms] OR "diet"[All Fields]) OR ("life style"[MeSH Terms] OR ("life"[All Fields] AND "style"[All Fields]) OR "life style"[All Fields] OR "lifestyle"[All Fields] OR "lifestyles"[All Fields]) OR "Lifestyle Interventions"[All Fields]) AND ("nepal"[MeSH Terms] OR "nepal"[All Fields] OR "nepal s"[All Fields]) AND ("diabetes mellitus"[MeSH Terms] OR ("diabetes"[All Fields] AND "mellitus"[All Fields]) OR "diabetes mellitus"[All Fields]) AND ("randomized controlled trial"[Publication Type] AND 2000/01/01:2024/12/31[Date - Publication])) AND ((ffrft[Filter]) AND (randomizedcontrolledtrial[Filter]))	2
Sri Lanka	(Exercise OR Diet OR Lifestyle OR "Lifestyle Interventions") AND "Sri Lanka" AND Diabetes Mellitus AND ((randomizedcontrolledtrial[Filter]) AND (2000:2024[pdat]))	(("exercise"[MeSH Terms] OR "exercise"[All Fields] OR "exercises"[All Fields] OR "exercise therapy"[MeSH Terms] OR ("exercise"[All Fields] AND "therapy"[All Fields]) OR "exercise therapy"[All Fields] OR "exercising"[All Fields] OR "exercise s"[All Fields] OR "exercised"[All Fields] OR "exerciser"[All Fields] OR "exercisers"[All Fields] OR ("diet"[MeSH Terms] OR "diet"[All Fields]) OR ("life style"[MeSH Terms] OR ("life"[All Fields] AND "style"[All Fields]) OR "life style"[All Fields] OR "lifestyle"[All Fields] OR "lifestyles"[All Fields]) OR "Lifestyle Interventions"[All Fields]) AND "Sri Lanka"[All Fields] AND ("diabetes mellitus"[MeSH Terms] OR ("diabetes"[All Fields] AND "mellitus"[All Fields]) OR "diabetes mellitus"[All Fields]) AND ("randomized controlled trial"[Publication Type] AND 2000/01/01:2024/12/31[Date - Publication])) AND ((ffrft[Filter]) AND (randomizedcontrolledtrial[Filter]))	9
Bangladesh	(Exercise OR Diet OR Lifestyle OR "Lifestyle Interventions") AND Bangladesh AND Diabetes Mellitus AND ((randomizedcontrolledtrial[Filter]) AND (2000:2024[pdat]))	(("exercise"[MeSH Terms] OR "exercise"[All Fields] OR "exercises"[All Fields] OR "exercise therapy"[MeSH Terms] OR ("exercise"[All Fields] AND "therapy"[All Fields]) OR "exercise therapy"[All Fields] OR "exercising"[All Fields] OR "exercise s"[All Fields] OR "exercised"[All Fields] OR "exerciser"[All Fields] OR "exercisers"[All Fields] OR ("diet"[MeSH Terms] OR "diet"[All Fields]) OR ("life style"[MeSH Terms] OR ("life"[All Fields] AND "style"[All Fields]) OR "life style"[All Fields] OR "lifestyle"[All Fields] OR "lifestyles"[All Fields]) OR "Lifestyle Interventions"[All Fields]) AND ("bangladesh"[MeSH Terms] OR "bangladesh"[All Fields] OR "bangladesh s"[All Fields]) AND ("diabetes mellitus"[MeSH Terms] OR ("diabetes"[All Fields] AND "mellitus"[All Fields]) OR "diabetes mellitus"[All Fields]) AND ("randomized controlled trial"[Publication Type] AND 2000/01/01:2024/12/31[Date - Publication])) AND ((ffrft[Filter]) AND (randomizedcontrolledtrial[Filter]))	5
Pakistan	(Exercise OR Diet OR Lifestyle OR "Lifestyle Interventions") AND Pakistan AND Diabetes Mellitus AND ((randomizedcontrolledtrial[Filter]) AND (2000:2024[pdat]))	(("exercise"[MeSH Terms] OR "exercise"[All Fields] OR "exercises"[All Fields] OR "exercise therapy"[MeSH Terms] OR ("exercise"[All Fields] AND "therapy"[All Fields]) OR "exercise therapy"[All Fields] OR "exercising"[All Fields] OR "exercise s"[All Fields] OR "exercised"[All Fields] OR "exerciser"[All Fields] OR "exercisers"[All Fields] OR ("diet"[MeSH Terms] OR "diet"[All Fields]) OR ("life style"[MeSH Terms] OR ("life"[All Fields] AND "style"[All Fields]) OR "life style"[All Fields] OR "lifestyle"[All Fields] OR "lifestyles"[All Fields]) OR "Lifestyle Interventions"[All Fields]) AND ("pakistan"[MeSH Terms] OR "pakistan"[All Fields] OR "pakistan s"[All Fields]) AND ("diabetes mellitus"[MeSH Terms] OR ("diabetes"[All Fields] AND "mellitus"[All Fields]) OR "diabetes mellitus"[All Fields]) AND ("randomized controlled trial"[Publication Type] AND 2000/01/01:2024/12/31[Date - Publication])) AND ((ffrft[Filter]) AND (randomizedcontrolledtrial[Filter]))	17
India	(Exercise OR Diet OR Lifestyle OR "Lifestyle Interventions") AND India AND Diabetes Mellitus AND ((randomizedcontrolledtrial[Filter]) AND (2000:2024[pdat]))	(("exercise"[MeSH Terms] OR "exercise"[All Fields] OR "exercises"[All Fields] OR "exercise therapy"[MeSH Terms] OR ("exercise"[All Fields] AND "therapy"[All Fields]) OR "exercise therapy"[All Fields] OR "exercising"[All Fields] OR "exercise s"[All Fields] OR "exercised"[All Fields] OR "exerciser"[All Fields] OR "exercisers"[All Fields] OR ("diet"[MeSH Terms] OR "diet"[All Fields]) OR ("life style"[MeSH Terms] OR ("life"[All Fields] AND "style"[All Fields]) OR "life style"[All Fields] OR "lifestyle"[All Fields] OR "lifestyles"[All Fields]) OR "Lifestyle Interventions"[All Fields]) AND ("india"[MeSH Terms] OR "india"[All Fields] OR "india s"[All Fields] OR "indias"[All Fields]) AND ("diabetes mellitus"[MeSH Terms] OR ("diabetes"[All Fields] AND "mellitus"[All Fields]) OR "diabetes mellitus"[All Fields]) AND ("randomized controlled trial"[Publication Type] AND	57

		2000/01/01:2024/12/31[Date - Publication])) AND ((ffrft[Filter]) AND (randomizedcontrolledtrial[Filter]))	
		**Total Results - 90**	

Supplementary Table S2 : Web Of Science Search Strategy

Country	Search Query	Results
India	ALL=((Exercise OR Diet OR Lifestyle OR "Lifestyle Interventions") AND India AND Diabetes Mellitus AND (RCT or "Randomized Controlled Trial")) and Open Access Timespan: 2000-01-01 to 2024-04-24	47
Pakistan	ALL=((Exercise OR Diet OR Lifestyle OR "Lifestyle Interventions") AND Pakistan AND Diabetes Mellitus AND (RCT or "Randomized Controlled Trial")) and Open Access and Open Access Timespan: 2000-01-01 to 2024-04-24	14
Nepal	ALL=((Exercise OR Diet OR Lifestyle OR "Lifestyle Interventions") AND Nepal AND Diabetes Mellitus AND (RCT or "Randomized Controlled Trial")) and Open Access and Open Access Timespan: 2000-01-01 to 2024-04-24	3
Bangladesh	ALL=((Exercise OR Diet OR Lifestyle OR "Lifestyle Interventions") AND Bangladesh AND Diabetes Mellitus AND (RCT or "Randomized Controlled Trial")) and Open Access and Open Access and Open Access Timespan: 2000-01-01 to 2024-04-24	4
Sri Lanka	ALL=((Exercise OR Diet OR Lifestyle OR "Lifestyle Interventions") AND Sri lanka AND Diabetes Mellitus AND (RCT or "Randomized Controlled Trial")) and Open Access and Open Access and Open Access and Open Access Timespan: 2000-01-01 to 2024-04-24	9
Bhutan	ALL=((Exercise OR Diet OR Lifestyle OR "Lifestyle Interventions") AND Bhutan AND Diabetes Mellitus AND (RCT or "Randomized Controlled Trial")) and Open Access and Open Access and Open Access and Open Access Timespan: 2000-01-01 to 2024-04-24	0
Maldives	ALL=((Exercise OR Diet OR Lifestyle OR "Lifestyle Interventions") AND maldives AND Diabetes Mellitus AND (RCT or "Randomized Controlled Trial")) and Open Access and Open Access and Open Access and Open Access Timespan: 2000-01-01 to 2024-04-24	1
	**Total Results - 78**	

Supplementary Table S3: Scopus search Strategy

Country	Search Query	Results
Maldives	(TITLE-ABS-KEY (exercise OR diet OR lifestyle OR "lifestyle interventions") AND (maldives) AND (diabetes mellitus)) AND (LIMIT-TO (PUBYEAR , 2000 2024)) AND (LIMIT-TO (DOCUMENT_TYPE , "article"))	0
Bhutan	(TITLE-ABS-KEY (exercise OR diet OR lifestyle OR "lifestyle interventions") AND (bhutan) AND (diabetes mellitus)) AND (LIMIT-TO (PUBYEAR , 2000 2024)) AND (LIMIT-TO (DOCUMENT_TYPE , "article"))	0
Nepal	(TITLE-ABS-KEY (exercise OR diet OR lifestyle OR "lifestyle interventions") AND (nepal) AND (diabetes mellitus)) AND (LIMIT-TO (PUBYEAR , 2000 2024)) AND (LIMIT-TO (DOCUMENT_TYPE , "article"))	4
Sri Lanka	(TITLE-ABS-KEY (exercise OR diet OR lifestyle OR "lifestyle interventions") AND ("sri lanka") AND (diabetes mellitus)) AND (LIMIT-TO (PUBYEAR , 2000 2024)) AND (LIMIT-TO (DOCUMENT_TYPE , "article"))	3
Bangladesh	(TITLE-ABS-KEY (exercise OR diet OR lifestyle OR "lifestyle interventions") AND (bangladesh) AND (diabetes mellitus)) AND (LIMIT-TO (PUBYEAR , 2000 2024)) AND (LIMIT-TO (DOCUMENT_TYPE , "article"))	4
Pakistan	(TITLE-ABS-KEY (exercise OR diet OR lifestyle OR "lifestyle interventions") AND (pakistan) AND (diabetes mellitus)) AND (LIMIT-TO (PUBYEAR , 2000 2024)) AND (LIMIT-TO (DOCUMENT_TYPE , "article"))	9
India	(TITLE-ABS-KEY (exercise OR diet OR lifestyle OR "lifestyle interventions") AND (india) AND (diabetes mellitus)) AND (LIMIT-TO (PUBYEAR , 2000 2024)) AND (LIMIT-TO (DOCUMENT_TYPE , "article"))	43
	**Total Results - 63**	

Supplementary Table S4: CINAHL search Strategy (In EBSCOHost)

Country	Search query	Results
Maldives	(TX (exercise OR diet OR lifestyle OR "lifestyle interventions") AND (TX maldives) AND (TX diabetes mellitus)) AND (PY 2000:2024)	0
Bhutan	(TX (exercise OR diet OR lifestyle OR "lifestyle interventions") AND (TX bhutan) AND (TX diabetes mellitus)) AND (PY 2000:2024)	0
Nepal	(TX (exercise OR diet OR lifestyle OR "lifestyle interventions") AND (TX nepal) AND (TX diabetes mellitus)) AND (PY 2000:2024)	0
Sri Lanka	(TX (exercise OR diet OR lifestyle OR "lifestyle interventions") AND (TX "sri lanka") AND (TX diabetes mellitus)) AND (PY 2000:2024)	4
Bangladesh	(TX (exercise OR diet OR lifestyle OR "lifestyle interventions") AND (TX bangladesh) AND (TX diabetes mellitus)) AND (PY 2000:2024)	2
Pakistan	(TX (exercise OR diet OR lifestyle OR "lifestyle interventions") AND (TX pakistan) AND (TX diabetes mellitus)) AND (PY 2000:2024)	4
India	(TX (exercise OR diet OR lifestyle OR "lifestyle interventions") AND (TX india) AND (TX diabetes mellitus)) AND (PY 2000:2024)	19
	**Total Results - 29**	

Supplementary Table S5: Cochrane Library Search Strategy

Country	Search Query	Results

Maldives	((Exercise OR Diet OR Lifestyle OR "Lifestyle Interventions") AND ("Maldives") AND "Diabetes Mellitus")	0
Bhutan	((Exercise OR Diet OR Lifestyle OR "Lifestyle Interventions") AND ("Bhutan") AND "Diabetes Mellitus")	0
Nepal	((Exercise OR Diet OR Lifestyle OR "Lifestyle Interventions") AND ("Nepal") AND "Diabetes Mellitus")	0
Sri Lanka	((Exercise OR Diet OR Lifestyle OR "Lifestyle Interventions") AND ("Sri Lanka") AND "Diabetes Mellitus")	1
Bangladesh	((Exercise OR Diet OR Lifestyle OR "Lifestyle Interventions") AND ("Bangladesh") AND "Diabetes Mellitus")	1
Pakistan	((Exercise OR Diet OR Lifestyle OR "Lifestyle Interventions") AND ("Pakistan") AND "Diabetes Mellitus")	3
India	((Exercise OR Diet OR Lifestyle OR "Lifestyle Interventions") AND ("India") AND "Diabetes Mellitus")	30
		**Total Results - 35**

Supplementary Table S6: Summary Results of database search and full-text screening

**Database Search Results**	**Number of Titles**
PubMed	90
Web of Science	78
Scopus	63
CINAHL	29
Cochrane Library	35
Total titles	295
Duplicated Removed	96
Title and Abstract Screening	199
Records Excluded after Title/Abstract Screening	144
Selected and available for Full-text screening	55
Excluded during Full-text screening	41
Reasons for Exclusion	Different Study Population (5)<br>Different Study Outcomes (4)<br>Different study type (18)<br>Different Exposures (9)<br>Protocols (1)<br>Insufficient Information (4)
Finally Selected	14
Records identified from citation & reference search of eligible articles	4
Reports not retrieved	2
Reports retrieved and accessed for eligibility	2
Reports excluded	0
Studies included in the Review	16

Inclusion and exclusion criteria

Inclusion criteria:

I. **Population**:
- Adults aged 18 years or older
- Diagnosed with type 2 diabetes mellitus (T2DM)
- Participants from South Asian countries (e.g., India, Pakistan, Bangladesh, Sri Lanka, Nepal, Bhutan, Maldives)

II. **Intervention**:
- Studies involving lifestyle modifications such as physical activity and/or dietary changes.

III. **Comparison**:
- Randomized controlled trials (RCTs) with a comparison group (e.g., standard care, no intervention)

IV. **Outcome**:
- HbA1c levels and/or fasting blood glucose (FBG) levels

Publication type: RCT
Date of publication: January 1, 2000 to June 13, 2024
Language: English

Exclusion criteria:

Studies were excluded if:
- Studies with participants who are not diagnosed with T2DM
- Studies with participants under the age of 18 years
- Studies including pregnant or lactating women
- Studies that are not published in English

The difference between the protocol and review

We initially intended to measure additional outcomes, such as weight, BMI, and other anthropometric measures in addition to HbA1c and FBG. However, we found that most studies did not report these outcomes. Given that our primary focus is on glycemic outcomes, which are adequately represented by HbA1c and/or FBG, we chose not to exclude studies that lacked data on these additional outcomes. Additionally, while assessing publication bias, we encountered a limitation with Egger's test, which requires a minimum of 10 studies. Since our FBG data included only 9 studies, we decided to include Begg's test, even though it was not specified in our protocol. This adjustment was made to ensure a thorough analysis.

Sub-group analyses for HbA1c

Sensitivity Analysis

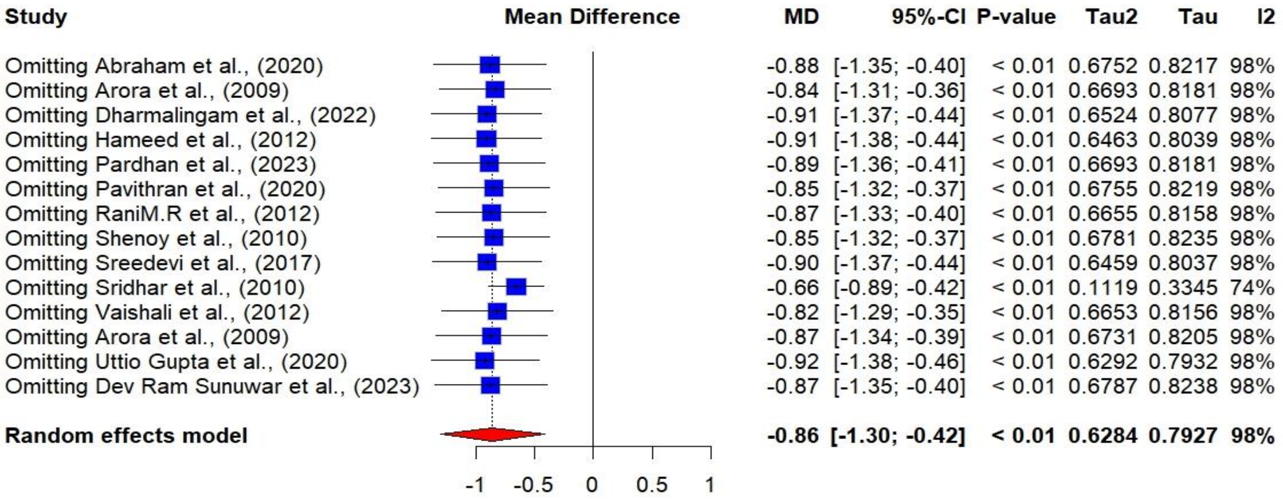

Supplementary Figure S1: Sensitivity Analyses for HbA1c by performing the meta-analysis by excluding one study at a time

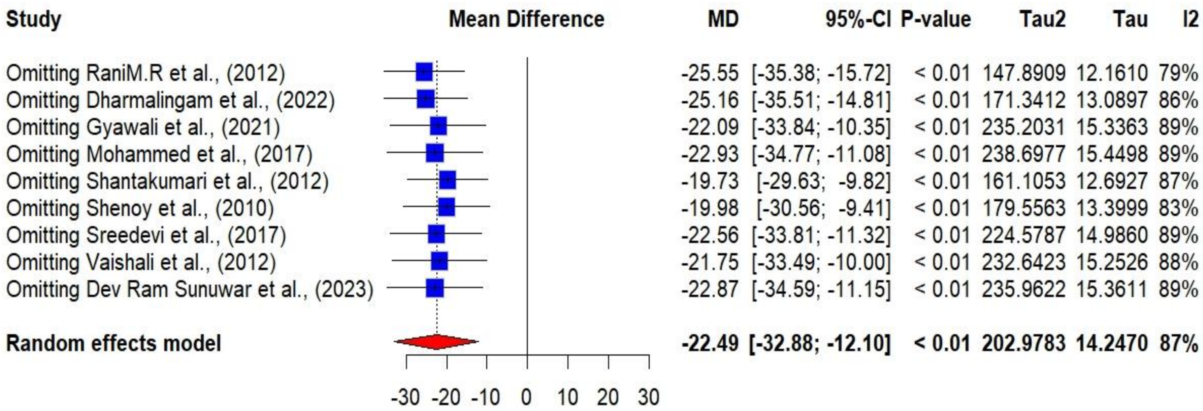

Supplementary Figure S2: Sensitivity Analyses for FBG by performing the meta-analysis by excluding one study at a time

Publication Bias Analysis

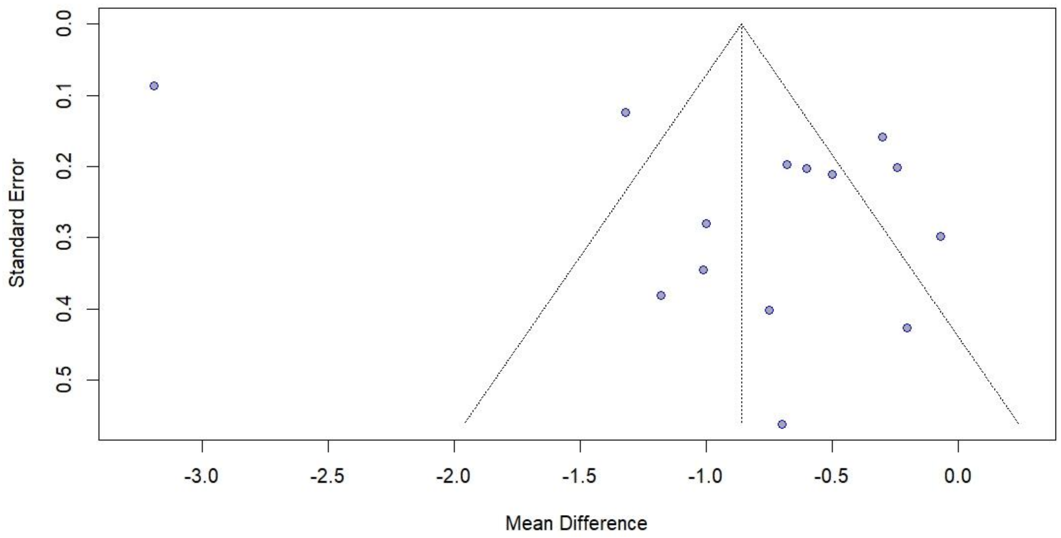

Supplementary Figure S3: Funnel plot for random effects meta-analysis of post intervention mean difference of HbA1C Levels

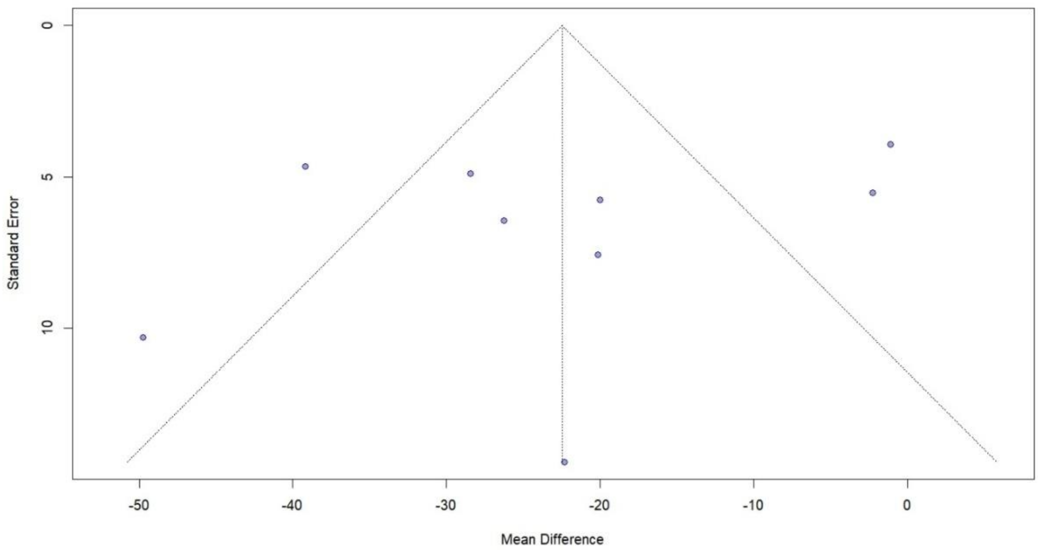

Supplementary Figure S4: Funnel plot for random effects meta-analysis of post intervention mean difference of FBG Levels

Trim & Fill method

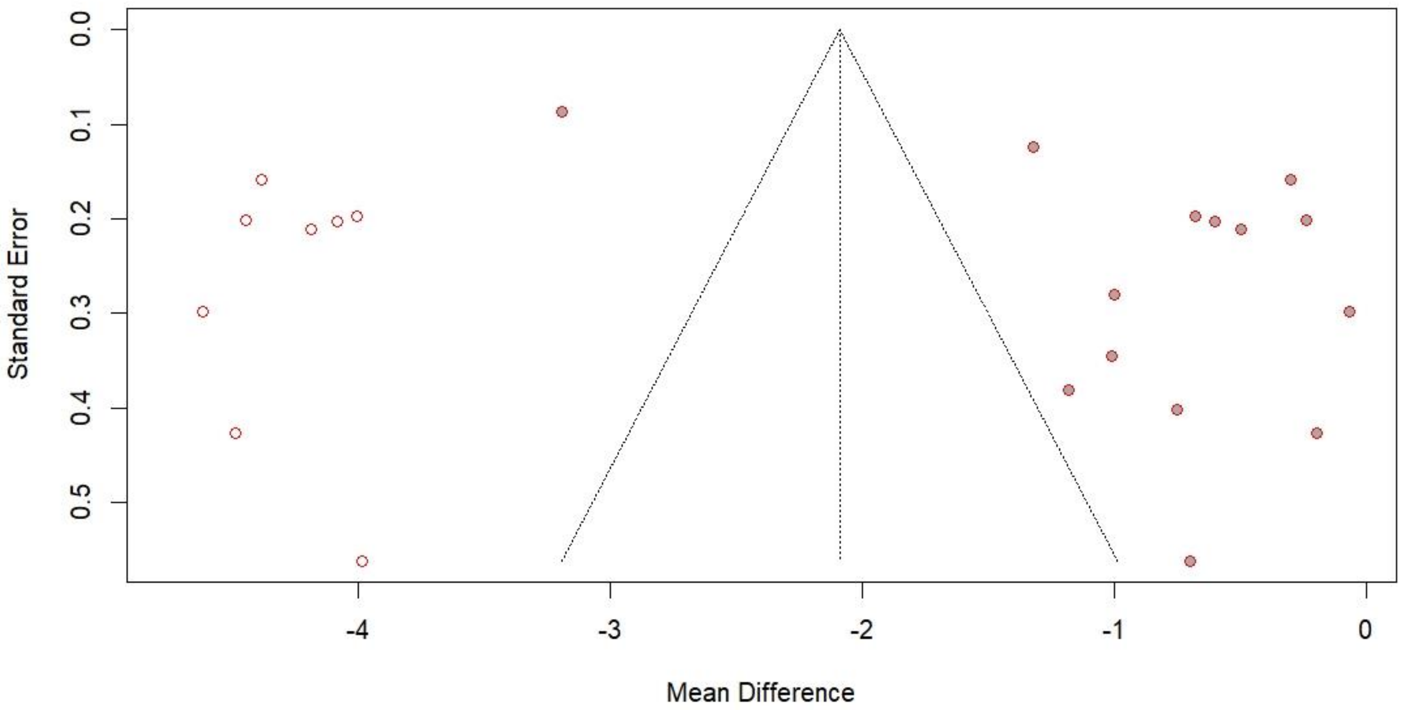

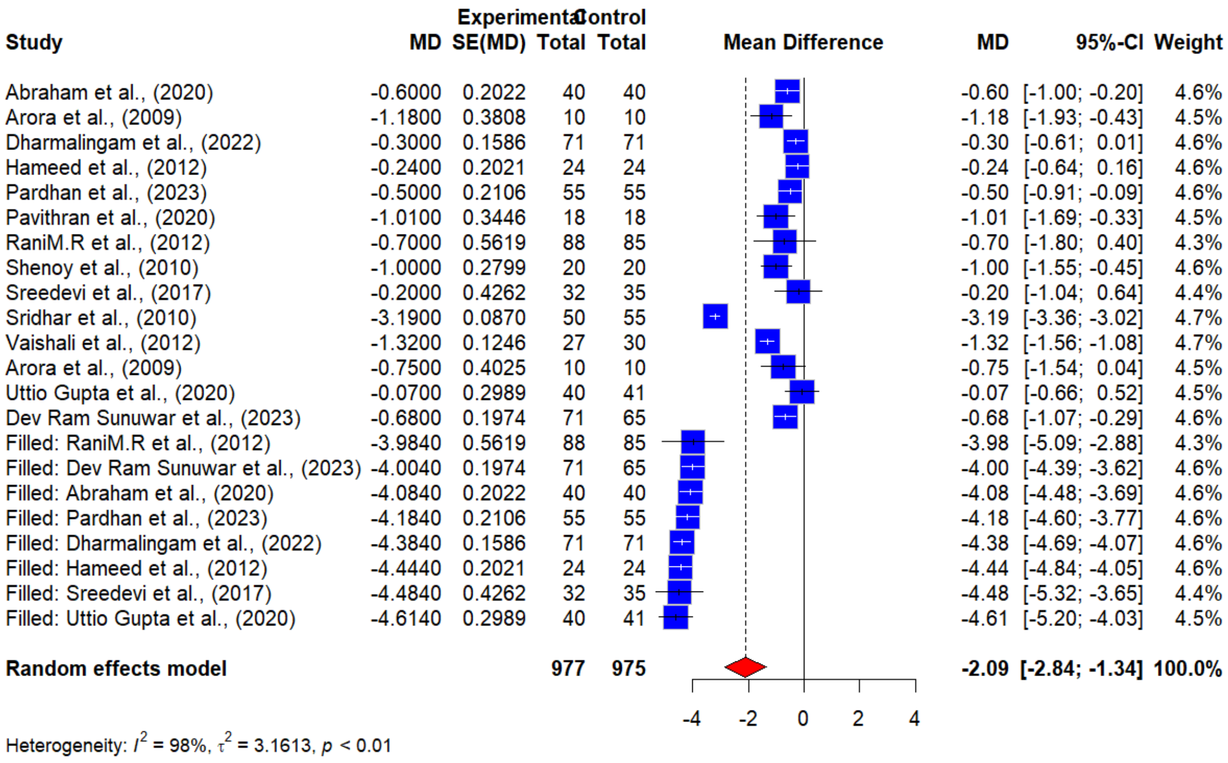

Supplementary Figure S5: Funnel and Forest plots Publication Bias Analysis using Trim and Fill method for mean difference of HbA1c levels

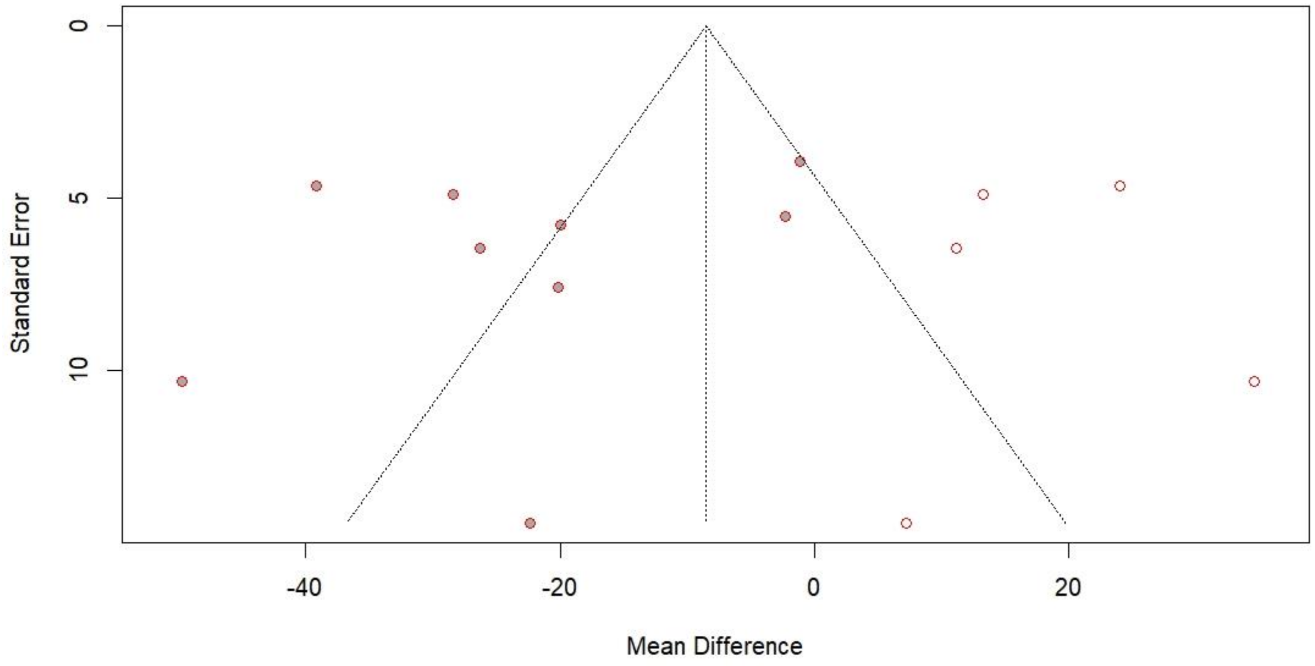

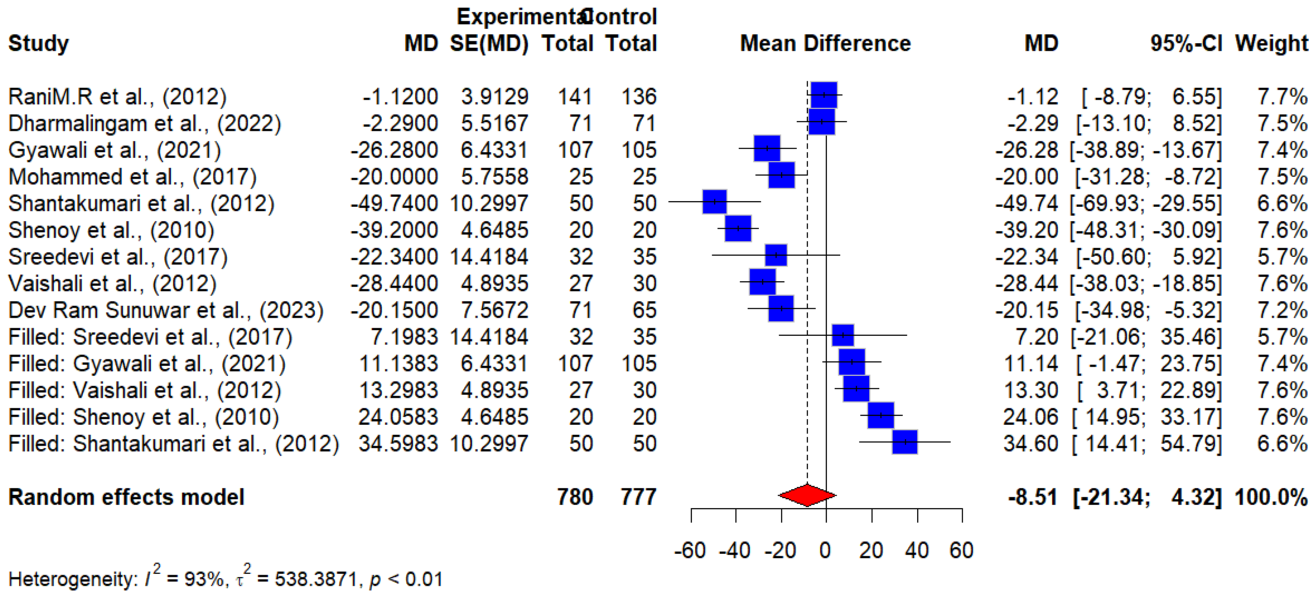

Supplementary Figure S6: Funnel and Forest plots of Publication Bias Analysis using Trim and Fill method for mean difference of FBG levels

PRISMA 2020 Checklist

Section and Topic	Item #	Checklist item	Location where item is reported
**TITLE**			
Title	1	Identify the report as a systematic review.	1
**ABSTRACT**			
Abstract	2	See the PRISMA 2020 for Abstracts checklist.	1,2
**INTRODUCTION**			
Rationale	3	Describe the rationale for the review in the context of existing knowledge.	2,3
Objectives	4	Provide an explicit statement of the objective(s) or question(s) the review addresses.	3
**METHODS**			
Eligibility criteria	5	Specify the inclusion and exclusion criteria for the review and how studies were grouped for the syntheses.	3,4
Information sources	6	Specify all databases, registers, websites, organisations, reference lists and other sources searched or consulted to identify studies. Specify the date when each source was last searched or consulted.	3
Search strategy	7	Present the full search strategies for all databases, registers and websites, including any filters and limits used.	3
Selection process	8	Specify the methods used to decide whether a study met the inclusion criteria of the review, including how many reviewers screened each record and each report retrieved, whether they worked independently, and if applicable, details of automation tools used in the process.	3
Data collection process	9	Specify the methods used to collect data from reports, including how many reviewers collected data from each report, whether they worked independently, any processes for obtaining or confirming data from study investigators, and if applicable, details of automation tools used in the process.	3
Data items	10a	List and define all outcomes for which data were sought. Specify whether all results that were compatible with each outcome domain in each study were sought (e.g. for all measures, time points, analyses), and if not, the methods used to decide which results to collect.	4
	10b	List and define all other variables for which data were sought (e.g. participant and intervention characteristics, funding sources). Describe any assumptions made about any missing or unclear information.	3, 4
Study risk of bias assessment	11	Specify the methods used to assess risk of bias in the included studies, including details of the tool(s) used, how many reviewers assessed each study and whether they worked independently, and if applicable, details of automation tools used in the process.	3
Effect measures	12	Specify for each outcome the effect measure(s) (e.g. risk ratio, mean difference) used in the synthesis or presentation of results.	4
Synthesis methods	13a	Describe the processes used to decide which studies were eligible for each synthesis (e.g. tabulating the study intervention characteristics and comparing against the planned groups for each synthesis (item #5)).	3
	13b	Describe any methods required to prepare the data for presentation or synthesis, such as handling of missing summary statistics, or data conversions.	4
	13c	Describe any methods used to tabulate or visually display results of individual studies and syntheses.	4
	13d	Describe any methods used to synthesize results and provide a rationale for the choice(s). If meta-analysis was performed, describe the model(s), method(s) to identify the presence and extent of statistical heterogeneity, and software package(s) used.	4
	13e	Describe any methods used to explore possible causes of heterogeneity among study results (e.g. subgroup analysis, meta-regression).	4
	13f	Describe any sensitivity analyses conducted to assess robustness of the synthesized results.	4

Reporting bias assessment	14	Describe any methods used to assess risk of bias due to missing results in a synthesis (arising from reporting biases).	4
Certainty assessment	15	Describe any methods used to assess certainty (or confidence) in the body of evidence for an outcome.	4
**RESULTS**			
Study selection	16a	Describe the results of the search and selection process, from the number of records identified in the search to the number of studies included in the review, ideally using a flow diagram.	5
	16b	Cite studies that might appear to meet the inclusion criteria, but which were excluded, and explain why they were excluded.	
Study characteristics	17	Cite each included study and present its characteristics.	7,8,9
Risk of bias in studies	18	Present assessments of risk of bias for each included study.	5,6
Results of individual studies	19	For all outcomes, present, for each study: (a) summary statistics for each group (where appropriate) and (b) an effect estimate and its precision (e.g. confidence/credible interval), ideally using structured tables or plots.	10, 11
Results of syntheses	20a	For each synthesis, briefly summarise the characteristics and risk of bias among contributing studies.	10
	20b	Present results of all statistical syntheses conducted. If meta-analysis was done, present for each the summary estimate and its precision (e.g. confidence/credible interval) and measures of statistical heterogeneity. If comparing groups, describe the direction of the effect.	10,11
	20c	Present results of all investigations of possible causes of heterogeneity among study results.	11-15
	20d	Present results of all sensitivity analyses conducted to assess the robustness of the synthesized results.	15
Reporting biases	21	Present assessments of risk of bias due to missing results (arising from reporting biases) for each synthesis assessed.	16
Certainty of evidence	22	Present assessments of certainty (or confidence) in the body of evidence for each outcome assessed.	15-16
**DISCUSSION**			
Discussion	23a	Provide a general interpretation of the results in the context of other evidence.	16
	23b	Discuss any limitations of the evidence included in the review.	17,18
	23c	Discuss any limitations of the review processes used.	17,18
	23d	Discuss implications of the results for practice, policy, and future research.	18
**OTHER INFORMATION**			
Registration and protocol	24a	Provide registration information for the review, including register name and registration number, or state that the review was not registered.	3
	24b	Indicate where the review protocol can be accessed, or state that a protocol was not prepared.	3
	24c	Describe and explain any amendments to information provided at registration or in the protocol.	Supp. Page 13
Support	25	Describe sources of financial or non-financial support for the review, and the role of the funders or sponsors in the review.	18
Competing interests	26	Declare any competing interests of review authors.	18
Availability of data, code and other materials	27	Report which of the following are publicly available and where they can be found: template data collection forms; data extracted from included studies; data used for all analyses; analytic code; any other materials used in the review.	18

From: Page MJ, McKenzie JE, Bossuyt PM, Boutron I, Hoffmann TC, Mulrow CD, et al. The PRISMA 2020 statement: an updated guideline for reporting systematic reviews. BMJ 2021;372:n71. doi: 10.1136/bmj.n71. This work is licensed under CC BY 4.0. To view a copy of this license, visit https://creativecommons.org/licenses/by/4.0/
